# Therapeutic outcomes of customized 3D-printed ankle-foot orthoses in children with spastic cerebral palsy: a case series study

**DOI:** 10.3389/fped.2025.1661098

**Published:** 2025-11-17

**Authors:** Cong Qin, Xiang Luo, Yan Yang, Yongrong Lun, Shali Yang, Lingfeng Chen

**Affiliations:** 1 Department of Pediatric Rehabilitation and Healthcare, Guigang People’s Hospital, Guigang, Guangxi, China; 2 Department of Hand and Foot Microsurgery and Orthopedics, Guigang People’s Hospital, Guigang, Guangxi, China

**Keywords:** 3D printing, ankle and foot orthosis, spastic cerebral palsy, cerebral palsy, ankle-foot orthoses

## Abstract

**Objective:**

To retrospectively evaluate the therapeutic effects of 3D-printed ankle-foot orthoses in children with spastic cerebral palsy (SPC).

**Methods:**

We conducted a retrospective cohort study at Guigang People's Hospital, reviewing medical records of children diagnosed with SPC between January 2022 and June 2024. A total of 124 patients who met the inclusion criteria were divided into two groups based on the orthotic device they received: the treatment group (*n* = 62) used 3D-printed orthoses (aluminum alloy uppers with TPU soles), while the control group (*n* = 62) used traditional polyethylene orthoses. All patients underwent standardized rehabilitation training. Clinical outcomes including passive ankle dorsiflexion, Gross Motor Function Measure (GMFM) scores, and gait parameters (step length, width, and cadence) were assessed based on follow-up records at 3 months after orthotic use.

**Results:**

Baseline data showed no significant differences between the two groups (*P* > 0.05). At the 3-months follow-up, both groups demonstrated functional improvements, with the treatment group exhibiting significantly greater gains. The passive ankle dorsiflexion angle in the treatment group was significantly lower (88.07 ± 3.18 degrees) than the control group (90.08 ± 2.65 degrees, *P* = 0.027). Post-treatment GMFM scores were significantly higher in the treatment group (74.98 ± 3.42 points) compared to the control group (69.08 ± 2.95 points, *P* = 0.001). The treatment group also showed significantly greater improvements in step length (increasing to 31.15 ± 4.18 vs. 28.68 ± 4.32 cm in control, *P* = 0.01), step speed (increasing to 0.56 ± 010 vs. 0.52 ± 0.12 m/s in control, *P* = 0.022), and reduced step width (decreasing to 14.52 ± 2.36 vs. 15.82 ± 2.40 cm in control, *P* = 0.011). The 3D-printed AFOs were significantly lighter (123.6 ± 36.15 g) and thinner (1.71 ± 0.17 mm) than the traditional AFOs (183.2 ± 65.78 g and 3.00 mm, *P* < 0.001).

**Conclusion:**

This retrospective study suggests that 3D-printed ankle-foot orthoses may offer improved comfort, durability, and functional benefits in gait performance among children with SPC compared to conventional orthoses.

## 1 Introduction

Cerebral palsy (CP) is a permanent disorder of movement and posture resulting from abnormal brain development or injury during childhood (1). Among the various types of CP, spastic cerebral palsy (SPC) is the most common, characterized by persistent muscle spasms and impaired coordination, usually accompanied by varying degrees of motor function limitations (2). To improve these children's walking ability and activities of daily living (ADL), Ankle-Foot Orthoses (AFOs) are important rehabilitation aids, which are mainly used to correct the deformity of feet and ankles, provide necessary support, and improve walking stability and sports efficiency (3, 4). AFOs are predominantly made from materials such as polyethylene, polypropylene, or low-temperature thermoplastic sheets. Although traditional AFO materials offer some level of processability and adaptability, they still face challenges such as excessive weight, poor breathability, and inadequate comfort during prolonged use (5, 6). Furthermore, the conventional manufacturing process of AFOs is complex, involving multiple manual operations and specialized equipment. This complexity makes it difficult to precisely adapt the orthosis to the patient's unique anatomical structure, resulting in suboptimal individualized adaptability and functionality. With advancements in technology, 3D printing has demonstrated unique advantages in the medical field, particularly in the manufacture of rehabilitation devices (7). 3D printing technology can accurately manufacture personalized rehabilitation aids based on the patient's specific anatomical data, which has the characteristics of flexible design, fast iteration speed and high material utilization rate (8). This technology allows for the use of variety materials in printing, such as lightweight aluminum alloy and thermoplastic polyurethane (TPU). The incorporation of these materials reduces the weight of the final product while enhancing comfort and breathability (9). In recent years, the application of 3D printing AFOs in children with SPC has increased. Through personalized design, 3D-printed AFOs not only provide the necessary mechanical support but also enable structural optimization, such as adjusting the orthosis thickness and incorporating ventilation holes. These adjustments help meet the physiological and functional needs of children at different stages of growth (10). However, the specific clinical efficacy and advantages of 3D-printed AFOs in children with SPC, particularly in comparison with traditional AFOs, still require systematic clinical research and evaluation (11).

Therefore, this study aims to systematically evaluate and compare the therapeutic effectiveness of 3D-printed AFOs vs. traditional AFOs in the rehabilitation of children with SPC through a retrospective comparative analysis. The assessment focuses on key functional indicators, including ankle joint mobility, Gross Motor Function Measure (GMFM) scores, and walking parameters. This research seeks to identify a more effective and comfortable orthotic strategy, thereby providing stronger scientific evidence and technical support for optimizing rehabilitation interventions in children with SPC.

## 2 Materials and methods

### 2.1 General information

From January 2022 to June 2024, a total of 124 children diagnosed with SPC were retrospectively included in this study based on their medical records at Guigang People's Hospital.

Inclusion criteria were as follows: (1) Diagnosed with SPC according to the 2022 *Guidelines for Rehabilitation of Cerebral Palsy in China* (6); (2) Aged between 3 and 6 years; (3) Gross motor function classified as grade I or II; (4) Availability of complete clinical and follow-up data.

Exclusion criteria included: (1) Severe ankle joint malformation or Achilles tendon contracture; (2) Presence of neuromuscular or musculoskeletal conditions affecting gait such as fixed joint contractures, significant hip pathology, or recent orthopedic surgery that could confound gait outcomes; (3) Cognitive impairments that precluded understanding of instructions, or language comprehension ability below stage 4-1 according to the S-S Children's Language Development Delay Test.

Based on the type of ankle-foot orthosis recorded in their medical records, participants were categorized into two groups: 62 children in the 3D-printed AFO group and 62 in the traditional polyethylene AFO group. Both groups received standardized rehabilitation training protocols during the treatment period.

### 2.2 Ethical considerations

The current study was approved by the Ethics Committee of the Guigang People's Hospital (Approval number: GPH202412181). Written informed consents from all patients were obtained in any experimental work with humans.

### 2.3 Manufacturing method of 3D printing orthosis

The orthoses were customized for each child based on a comprehensive initial assessment of their anatomical needs. This included a detailed evaluation of the child's foot shape, ankle range of motion, and gait patterns. 3D scanning technology was used to capture the child's foot structure, which was then imported into Computer-Aided Design (CAD) software for precise adjustments. The orthoses were designed to accommodate individual anatomical features such as foot arch height, ankle alignment, and any other specific requirements, such as toe alignment or pressure distribution. Pressure management was achieved by maintaining an optimal 2–3 mm clearance between the skin and the orthosis. Custom modifications were also made during the fitting process to ensure proper comfort and support for each child.

#### 2.3.1 Design of the plantar component of the orthosis

**Figure 1:**
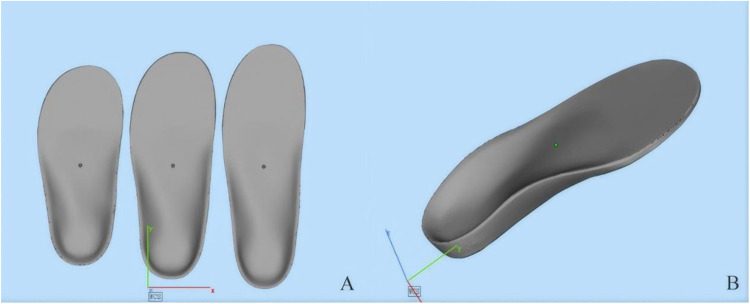
Three-dimensional digital models of custom orthopedic insoles. **(A)** Three insoles of varying sizes depicted in a frontal view, demonstrating scalability. **(B)** An angled view of a single insole, illustrating the anatomical contours and structural design.

According to clinical manufacturing records, a standardized database of orthopedic insole templates had been established prior to treatment, with data model files corresponding to various shoe sizes (Figure 1A). For each child, measurements such as foot length, forefoot width, hindfoot width, and arch height had been recorded. Based on these measurements, an insole template with a closely matching foot length was selected and subsequently adjusted according to the child's specific anatomical data (Figure 1B). The customized insole served as the basis for designing the bottom surface of the orthosis, aiming to provide a personalized fit and optimized functionality.

#### 2.3.2 Designing the upper component of the ankle-foot orthosis

According to clinical manufacturing records, a 3D scanner had been used to obtain detailed measurements of each child's calf. During the scanning process, the ankle joint had been maintained in a neutral position, with the foot rotated approximately 5 degrees to facilitate accurate data acquisition. The resulting STL files captured the three-dimensional geometry of the calf (Figure 1).

**Figure 2:**
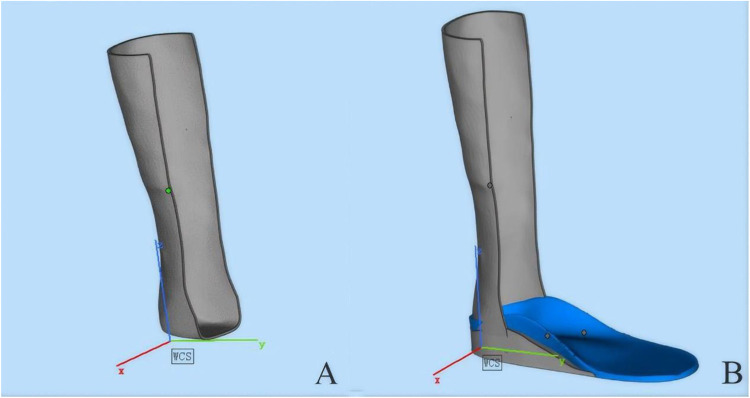
Sequential 3D renderings of an orthotic device assembly. **(A)** A preliminary structural framework of the device. **(B)** The integrated model featuring a blue footbed component. Cartesian axis markers (X, Y, Z) are included for spatial reference.

The shell data was subsequently processed using 3D design software. The shell thickness had been adjusted between 1.7 and 2.5 mm based on individual characteristics such as weight and muscle tone. To enhance comfort, excess material in the anterior region of the calf had been trimmed (Figure 2A). Finally, the customized upper shell was integrated with the pre-designed insole to assemble the static ankle-foot orthosis (Figure 2B). This customized orthotic design aimed to accommodate individual anatomical needs and promote improved rehabilitation outcomes.

#### 2.3.3 Optimization and design of dynamic ankle-foot orthosis

**Figure 3:**
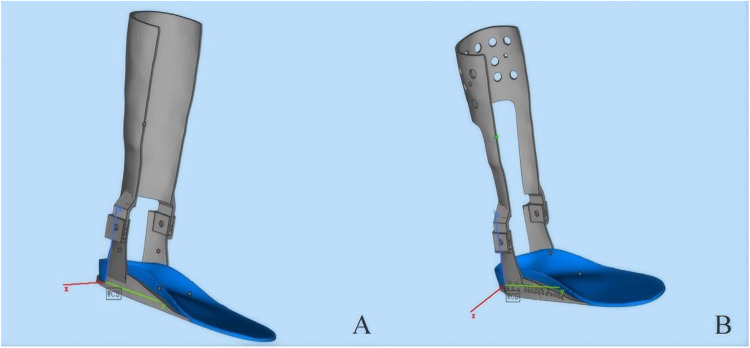
Comparative 3D models of a transtibial prosthetic limb. Both designs incorporate a gray shank and a blue footplate. **(A)** A prototype with a solid calf segment. **(B)** An advanced design featuring a perforated pattern to enhance ventilation and reduce weight.

According to available design documentation, the dynamic ankle-foot orthosis (AFO) had initially been developed based on a dynamic model, as shown in Figure 3A. For cases requiring dynamic ankle-foot orthoses, the height of the ankle joint rotation center had been manually recorded as the vertical distance from the lower edge of the medial malleolus to the ground. This model had been structured to provide active support and enable controlled ankle joint motion tailored to the rehabilitation needs of children with SPC. The finalized version of the design (Figure 3B) featured an optimized and streamlined structure that retained core mechanical stability and flexibility. These documented design enhancements aimed to reduce the device's weight and improve user comfort, while supporting gait stability and functional mobility during rehabilitation.

#### 2.3.4 3D printing process

**Figure 4:**
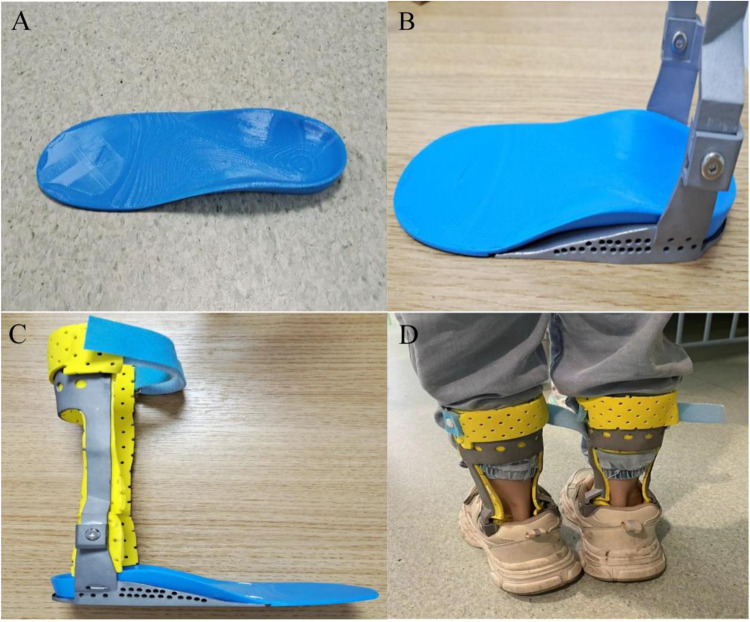
Prototype application and integration of the orthopedic system. **(A)** The custom-fabricated blue orthopedic insole. **(B)** The insole integrated into a foot brace with metallic supportive elements. **(C)** A lateral view of the fully assembled brace on a neutral background. **(D)** A clinical demonstration of the bilateral brace system worn with standard footwear.

According to the archived design and manufacturing records, prior to the printing phase, the scanned calf data were imported for a final compatibility check to ensure accurate alignment and fit. The clearance between the skin and the orthosis was measured at various angles, with an optimal gap of 2–3 mm maintained. This clearance facilitates the application of a protective inner liner, preventing localized pressure points that could cause discomfort or skin irritation. The insole component of the orthosis was fabricated using soft thermoplastic polyurethane material (TPU95A), chosen for its flexibility and comfort, while the leg portion was printed using aluminum alloy metal powder, selected for its strength and durability. The completed printed products are depicted in the following figures: Figure 4A: orthotic insole, Figure 4B: schematic diagram of the insole and leg combination, Figure 4C: finished product drawing, and Figure 4D: Orthosis worn with shoes.

### 2.4 Methods

#### 2.4.1 Comprehensive rehabilitation training

Based on the medical and rehabilitation records, children in the control group had used traditional ankle-foot orthoses, while those in the treatment group had been fitted with combined ankle-foot orthoses (a rigid aluminum alloy leg component and a soft, flexible TPU insole). According to documentation, both groups had received comprehensive rehabilitation therapy primarily consisting of exercise-based interventions, including stretching exercises, muscle strength training, balance training, and gait training. The recorded rehabilitation sessions were approximately 3 h per day, 5 days per week, and lasted for a total of 3 months.

The medical records further indicated that throughout the treatment period, group assignment, intervention delivery, and outcome assessments had been performed by different staff members. A single-blind protocol had been employed during the clinical process, as noted in the institutional documentation.

#### 2.4.2 Observation index

In this study, several key clinical indicators were retrospectively collected to evaluate the therapeutic outcomes of different orthotic interventions in children with CP. These indicators were extracted from medical and rehabilitation records to comprehensively assess motor function improvements, and included the following aspects:

1. Passive dorsiflexion angle of ankle joint was assessed with the child positioned in a supine posture. In this study, the angle was measured relative to the neutral position, which is defined as 90 degrees between the foot and the leg. During the measurement, the maximum passive dorsiflexion angle was determined without the involvement of active muscle activity. To ensure accuracy, it was verified that the ankle joint remained in a fully relaxed state throughout the assessment. This procedure was meticulously followed to obtain reliable and precise measurements of the ankle joint's passive mobility.
2. Gross motor function. Evaluation tool: The motor function of the children was assessed using the Gross Motor Function Measure-88 (GMFM-88) (12), specifically focusing on Domain D (standing) and Domain E (walking and jumping). The GMFM-88 is a widely recognized and validated scale for evaluating motor function in patients with CP, demonstrating high reliability and validity in clinical and research settings. Evaluation timing: Assessments were conducted both before and after the treatment period to evaluate the therapeutic effects of the interventions.
3. Evaluation of walking function. Evaluation method: Children were instructed to walk along a flat walkway measuring 10 m in length and 1 m in width, which was marked with scale lines [GMFM scores (Domain D and E), step length, step speed, and step width] to ensure accurate measurement. Prior to walking, the soles of the children's feet were coated with ink pads to capture their footprints. Evaluation timing: Evaluations were conducted at two distinct time points: before the initiation of treatment and 3 months following the completion of treatment.
4. Evaluation of Orthosis Physical Characteristics. Weight and thickness: The physical characteristics of the orthoses were assessed by measuring their weight (in grams) and thickness (in millimeters). Evaluating these parameters is essential for understanding how the physical configuration of the orthosis may affect the gait and motor function of children with SPC. Specifically, lighter and thinner orthoses are hypothesized to enhance mobility and comfort, potentially leading to more effective rehabilitation outcomes.

### 2.5 Statistical analysis

In this study, SPSS22.0 statistical software (IBM Corp., Armonk, NY, USA) was used to analyze the data. All quantized data were presented by the mean standard deviation. Baseline and post-treatment indicators, including ankle passive dorsiflexion angle, GMFM scores, and walking function parameters (such as step length, step speed, and step width), were compared between the two groups using independent samples *t*-tests. Categorical variables were analyzed using Chi-square tests. To ensure the validity of these parametric tests, the normality of all continuous variables was first assessed with the Shapiro–Wilk normality test. A significance level of *P* < 0.05 was established for all statistical comparisons. For data that did not meet the assumptions of normal distribution, nonparametric tests such as the Mann–Whitney *U* test were utilized to compare differences between the two groups. To assess changes in continuous variables within each group before and after treatment, paired sample *t*-tests were conducted. In cases of non-normal data distribution, the Wilcoxon signed-rank test was used. The significance level of *α* = 0.05 was set, with *P*-values below 0.05 indicating statistical differences. Additionally, Bonferroni correction was applied to adjust for multiple comparisons, reducing the likelihood of Type I errors.

## 3 Results

### 3.1 General information of patients

In this study, medical records of 124 children diagnosed with SPC and treated at our institution between January 2023 and June 2023 were reviewed. Based on the orthotic intervention recorded in the rehabilitation records, the children were categorized into two groups: the treatment group (*n* = 62), who wore combined AFOs manufactured using 3D printing technology, and the control group (*n* = 62), who wore traditional polyethylene orthoses.

General data of the two groups, including gender, age, classification of SPC and GMFCS were compared, and the results showed that there was no statistical significance in baseline data between the two groups (*P* > 0.05). In the treatment group, there were 40 males and 22 females, while the control group comprised 38 males and 24 females. The mean age of participants in the treatment group was 4.18 ± 1.1 years, compared to 4.06 ± 0.96 years in the control group. Regarding the type of CP, the treatment group included 51 cases of diplegia and 11 cases of quadriplegia, whereas the control group consisted of 49 cases of diplegia and 13 cases of quadriplegia. According to the Gross Motor Function Classification System (GMFCS) grading, the treatment group had 11 cases classified as Grade I, 49 cases as Grade II, and 3 cases as Grade III. In contrast, the control group included 17 cases of Grade I, 39 cases of Grade II, and 6 cases of Grade III. These baseline characteristics are summarized in Table 1.

**Table 1:** Comparison of two groups of general data.

Groups	Gender		Age (years)	Spastic types		GMFCS classification		
	Man	Woman		Diplegia	Quadriplegia	Level I	Level II	Level III
Control group (*n* = 62)	40	22	4.12 ± 1.1	49	13	17	39	6
Treatment group (*n* = 62)	38	24	4.08 ± 0.96	51	11	10	49	3
*t*	0.212		0.23	0.24		0.22		
*P*	0.56		0.62	0.53		0.612		

### 3.2 Comparison of passive dorsiflexion angle of ankle joint before and after treatment

Prior to treatment, there was no statistically significant difference in the passive dorsiflexion angle of the ankle joint between the treatment and control groups. Specifically, the treatment group had an average angle of 92.27 ± 4.30 degrees, while the control group averaged 91.78 ± 3.75 degrees (*P* = 0.728), indicating comparable baseline conditions.

**Figure 5:**
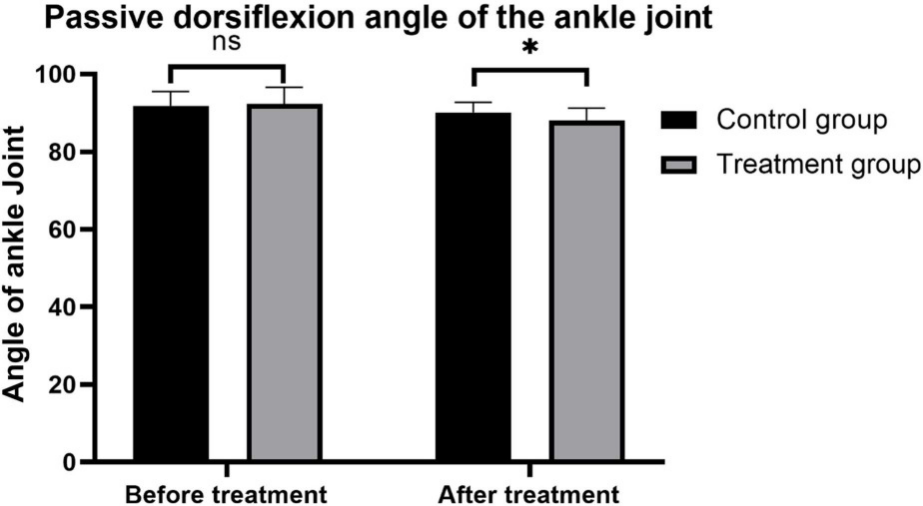
Comparison of passive dorsiflexion angles of the ankle joint before and after treatment between the two groups.

Compared to pre-intervention, both groups showed a reduction in the passive dorsiflexion angle of the ankle joint after 3 months of treatment, demonstrating the therapeutic effect of the interventions. The treatment group showed a decrease to an average angle of 88.07 ± 3.18 degrees, whereas the control group reduced to 90.08 ± 2.65 degrees. The post-treatment comparison revealed that the treatment group had a significantly lower passive dorsiflexion angle compared to the control group (*P* = 0.027), suggesting a more pronounced therapeutic effect in the treatment group. These findings are summarized in Table 2 and Figure 5.

**Table 2:** Passive dorsiflexion angle of the ankle joint before and after treatment $\left(\bar{x}\pm s\right)$.

Groups	Before treatment	After treatment	*t*	*P*
Control group (*n* = 62)	91.78 ± 3.75	90.08 ± 2.65	3.256	0.002
Treatment group (*n* = 62)	92.27 ± 4.30	88.07 ± 3.18	4.84	<0.001
*t*	0.284	2.403		
*P*	0.728	0.027		

### 3.3 GMFM scores between two groups of children

**Figure 6:**
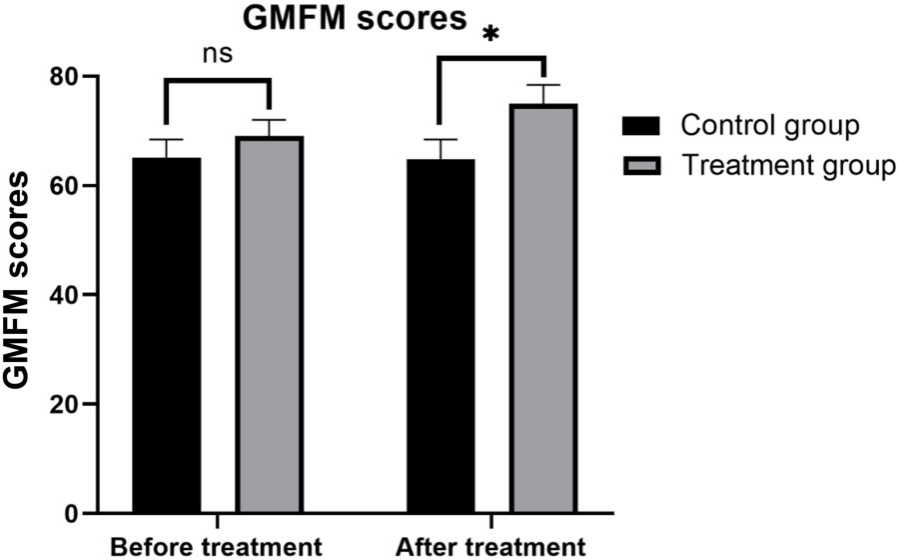
Comparison of GMFM (Gross Motor Function Measure) item scores before and after treatment between the two groups.

There was no significant difference in GMFM scores between the treatment and control groups before the initiation of treatment, indicating that the baseline motor functions were comparable (*P* > 0.05). Specifically, the treatment group had a mean GMFM score of 64.86 ± 3.54 points, while the control group had a mean score of 65.16 ± 3.28 points (*P* = 0.805). After 3 months of treatment, GMFM scores significantly improved in both groups, demonstrating the effectiveness of both types of orthoses in enhancing gross motor function in children with SPC. Notably, the treatment group exhibited a more substantial improvement, with post-treatment GMFM scores increasing to 74.98 ± 3.42 points, compared to 69.08 ± 2.95 points in the control group. The difference in post-treatment GMFM scores between the two groups was statistically significant (*P* = 0.001), suggesting that 3D-printed AFOs are more effective than traditional orthoses in promoting gross motor function improvements. These findings are summarized in Table 3 and Figure 6.

**Table 3:** GMFM scores before and after treatment in the two groups $\left(\bar{x}\pm s\right)$.

Groups	Before treatment	After treatment	*t*	*P*
Control group (*n* = 62)	65.16 ± 3.28	69.08 ± 2.95	−4.013	<0.001
Treatment group (*n* = 62)	64.86 ± 3.54	74.98 ± 3.42	−5.329	<0.001
*t*	0.251	−3.933		
*P*	0.805	0.001		

### 3.4 Comparison of walking function between two groups of children

In the evaluation of walking function, three key indices were systematically compared: step length, step width, and step speed. Before treatment, there were no statistically significant differences in the initial values of these walking parameters between the treatment and control groups (*P* > 0.05), indicating comparable baseline walking functions. After 3 months of treatment, both groups demonstrated significant improvements in step length and step speed, along with a reduction in step width. Specifically, the step length of the treatment group increased from 24.30 ± 4.88 cm before treatment to 31.15 ± 4.18 cm, the step speed increased from 0.43 ± 0.06 to 0.56 ± 0.10 m/s, and the step width decreased from 18.38 ± 2.94 to 14.52 ± 2.36 cm. These changes were statistically significant (*P* < 0.001). Although the control group also demonstrated improvements in walking function, the magnitude of these changes was less pronounced compared to the treatment group. Specifically, the control group exhibited an increase in step length from 25.08 ± 5.02 to 28.68 ± 4.32 cm and an increase in step speed from 0.45 ± 0.08 to 0.52 ± 0.12 m/s. Additionally, there was a moderate decrease in step width from 17.62 ± 2.86 to 15.82 ± 2.40 cm. All these changes were statistically significant (*P* < 0.05).

**Figure 7:**
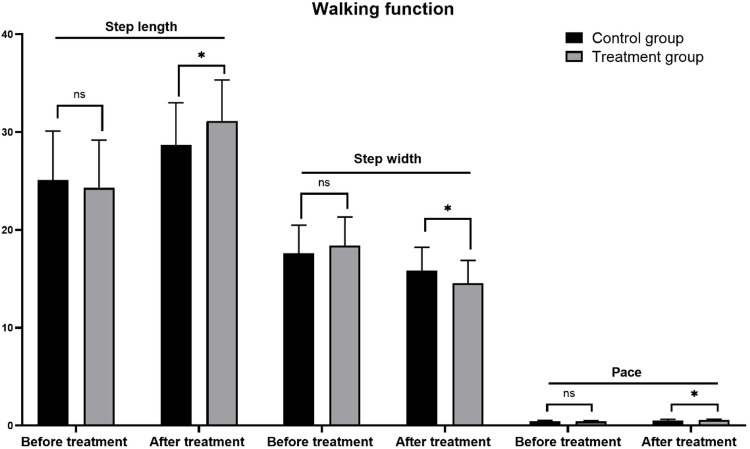
Comparison of walking function before and after treatment between the two groups.

In comparing the walking function between the two groups, the treatment group exhibited significantly greater improvements in step length and walking speed after the treatment period (step length: *P* = 0.01; walking speed: *P* = 0.022) compared to the control group. Additionally, the treatment group achieved a significantly narrower step width (*P* = 0.011) than the control group post-treatment. These findings demonstrate that the 3D-printed combined ankle-foot orthosis offers more substantial advantages in enhancing walking function in children with SPC. These results are detailed in Table 4 and Figure 7.

**Table 4:** Walking function between the two groups before and after treatment $\left(\bar{x}\pm s\right)$.

Groups	Step length		*t*	*P*	Step width		*t*	*P*	Pace		*t*	*P*
	Before treatment	After treatment			Before treatment	After treatment			Before treatment	After treatment		
Treatment group (*n* = 62)	24.30 ± 4.88	31.15 ± 4.18	7.302	0.000	18.38 ± 2.94	14.52 ± 2.36	6.992	0.000	0.43 ± 0.06	0.56 ± 0.10	6.623	0.000
Control group (*n* = 62)	25.08 ± 5.02	28.68 ± 4.32	3.886	00.000	17.62 ± 2.86	15.82 ± 2.40	3.325	0.001	0.45 ± 0.08	0.52 ± 0.12	3.091	0.002
*t*	0.816	2.618			1.272	2.586			1.152	2.334		
*P*	0.416	0.01			0.205	0.011			0.254	0.022		

### 3.5 Weight comparison of ankle-foot orthosis between two groups

**Figure 8:**
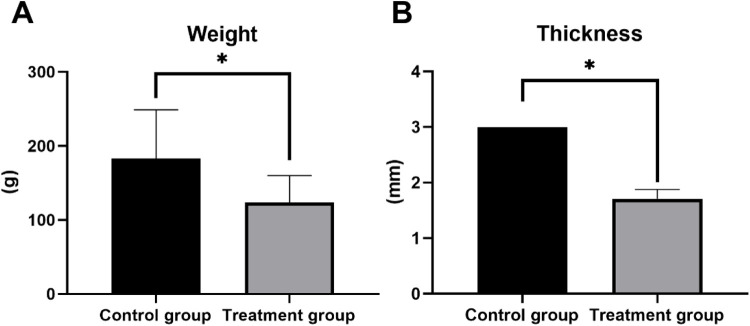
Quantitative comparison of material properties between control and treatment groups. (A) Bar graph comparing mean weight (g), showing a statistically significant reduction in the treatment group. (B) Bar graph comparing mean thickness (millimeters), showing a statistically significant reduction in the treatment group. (*p < 0.05, *p* < 0.01, *p < 0.001).

In this study, the 3D-printed AFOs demonstrated significantly different physical characteristics compared to traditional AFOs. Specifically, the average weight of the 3D-printed AFOs in treatment group was 123.6 ± 36.15 g, which was markedly lighter than the traditional AFOs in the control group's average weight of 183.2 ± 65.78 g. Statistical analysis confirmed that this weight difference was highly significant (*t* = 6.014, *P* < 0.001), indicating that 3D-printed AFOs are substantially lighter than their traditional counterparts. Similarly, when evaluating thickness, the 3D-printed AFOs had an average thickness of 1.71 ± 0.17 mm, whereas the traditional AFOs had an average thickness of 3.00 mm in thickness. The comparison of thickness between the two groups revealed a highly significant *t*-value of 23.45 with a corresponding *P*-value <0.001, underscoring that 3D-printing technology effectively produces thinner ankle-foot orthoses. These results are detailed in Table 5 and Figures 8A,B.

**Table 5:** Comparison of weight and thickness of AFOs in the two groups.

Groups	Control group (*n* = 62)	Treatment group (*n* = 62)	*t*	*P*
Weight (g)	183.2 ± 65.78	123.6 ± 36.15	6.014	0.000
Thickness (mm)	3.00	1.71 ± 0.17	23.45	0.000

## 4 Discussion

Children with CP often show equinus feet, varus or valgus feet due to the changes of muscle strength and muscle tension of lower limbs (13). AFOs are the most widely utilized orthotic devices in the management of children with CP (14). When properly fitted and worn, AFOs can significantly improve abnormal postures and correct foot and ankle deformities, thereby enhancing mobility and functional outcomes (15). This method requires extensive manual labor and specialized equipment, and it also generates considerable material waste, making the process less efficient and environmentally sustainable (16). In the traditional manufacturing method, the plaster bandage is usually taken from the mold first, then the plaster is poured into the mold and then the mold is manually repaired, and the orthosis is made of polymer materials. The manufacturing process is complicated, which requires gypsum bandage, gypsum scissors, gypsum powder, laser alignment instrument, vacuum pumping equipment, flat heater and other equipment, which requires high skills and experience of orthosis manufacturers (17, 18).

The results of this study indicate that, after treatment, the ankle dorsiflexion angle in the treatment group wearing 3D-printed AFOs was significantly lower than in the control group using traditional orthoses. This reduction suggests more effective correction of ankle positioning in the treatment group where the 3D-printed AFO forces the ankle into a more functionally neutral and stable position during the gait cycle compared to the traditional AFO. Additionally, the computer-aided design of the combined ankle-foot orthosis with hole patterns resulted in a lighter and more breathable product compared to traditional AFOs. These design improvements enhance wearing compliance by reducing the overall weight and increasing comfort, thereby facilitating better participation in activities such as standing, running, and jumping (19). Furthermore, the insoles of the 3D-printed AFOs were fabricated using soft thermoplastic materials, which allow for unrestricted plantar flexion at the tarsometatarsal joints of the forefoot. This flexibility allows for natural foot movements, promoting a more fluid and natural gait pattern. The arch support integrated into the design effectively corrects eversion and flatfoot deformities, providing stability to the foot during walking. This support not only aids children in pushing off the ground more efficiently but also significantly improves wearing comfort (20, 21). Similarly, a study by Wojciechowski et al. (7) demonstrated that 3D-printed AFOs for children with Charcot-Marie-Tooth disease offered improved biomechanics, such as normalizing ankle dorsiflexion. Notably, the redesigned 3D-printed AFOs were also lighter and provided better overall performance, further supporting the benefits of 3D printing in orthotic design.

Children with SPC require the ability to perform activities such as walking, running, and jumping, which necessitates orthotic support to manage the effects of gravity and spasticity. The combined ankle-foot orthosis, printed using aluminum alloy powder, offers high strength, low density, and lightweight properties. Thanks to the personalized design capabilities of 3D printing technology, the orthosis for children with CP, particularly those in middle and younger age groups, can be designed with a wall thickness as thin as 1.55 mm. This makes the orthosis nearly 50% thinner than traditional models, enhancing both comfort and functionality (22). Meanwhile, through structural optimization, non-functional parts are cut off and a large number of holes are made to facilitate air permeability, and the weight of the orthotics can be reduced by about 20%–30% compared with traditional orthotics (23). In terms of gross motor function training, the treatment group showed significant improvement, which facilitated better anti-gravity muscle strength training with improved sagittal and frontal plane alignment of the foot and ankle during functional activities. This enabled more significant improvement in gross motor skills, including standing, walking, and jumping exercises. In contrast, the control group's orthoses, while providing necessary lower limb support, were often bulky and rigid. This reduced their fit and comfort, making them less acceptable to children. 3D printing technology effectively addresses these challenges by allowing for the use of specialized materials, the creation of complex structures, and more precise processing, offering a more tailored and comfortable solution compared to traditional orthotics (17).

In addition, due to the fact that the forefoot is made of soft material, the movement of metatarsal joint is not restricted by orthosis. After treatment, the step length and pace of the treatment group are better than those of the control group, and the step width is smaller than that of the control group. The gait abnormalities of children with SPC are mainly characterized by small step length, slow pace, poor walking stability, and decreased walking distance and time. The walking stability is often maintained by increasing the step width and reducing the pace. Wearing combined ankle-foot orthosis can better improve children's walking ability, correct their abnormal walking posture and improve their walking comfort (24). In line with previous studies, our findings suggest that 3D-printed AFOs improve ankle mobility, and balance in children with SPC. For instance, a study by Cho et al. (25) reported similar improvements in gait parameters such as step length and stride width in stroke survivors using 3D-printed AFOs. Our study also aligns with a systematic review by Pollen et al. (26), which found that 3D-AFOs lead to significant improvements in gait biomechanics and patient satisfaction, supporting their efficacy as a rehabilitation tool.

In this study, the ankle-foot orthosis was made by 3D printing technology through layer-by-layer printing, which was made by “additive manufacturing”, with less loss of raw materials. AFOs are designed to be “simplified application and removal and easy maintenance” and typically require the use of materials such as plaster bandages and plaster molds during the casting process. The fabrication of these orthoses involves consuming plaster bandages and plaster mud to create the initial mold. Subsequently, the manufacturing process necessitates additional steps of cutting and grinding to shape the orthotic plates. The ankle-foot orthosis made of aluminum alloy by 3D printing has mature technology and simple design process. With the support of various powerful functions of 3D design software, the orthosis can be optimized in light weight and comfort, which is incomparable to traditional orthosis. The manufacturing process of traditional AFOs is inherently complex and labor-intensive, often requiring multiple manual steps and specialized materials. In contrast, the 3D printing process simplifies orthosis production by utilizing only a scanner, 3D designs software, and a compatible 3D printer.

The 3D ankle-foot orthosis designed and printed in a combined way has mature technology. Compared with the traditional manufacturing technology, 3D printing technology shortens the manufacturing time and simplify the manufacturing process of ankle-foot orthosis, and is easier to master and suitable for popularization and application compared with the traditional manufacturing method.

The ankle-foot orthosis can be customized to each patient's specific foot shape and medical condition using 3D printing with aluminum alloy powder. Aluminum alloy offers high strength, low density, lightweight, and thinness, which reduces the burden on children. This customization improves the orthosis's adaptability, enhances comfort, and alleviates pressure on the foot. It also helps correct joint deformities, prevent muscle atrophy, and supports multi-posture training for children with CP. Additionally, aluminum alloy meets the requirements for personalization and lightweight design, allowing for rapid manufacturing with high precision. However, this study did not include a mechanical analysis of the ankle and foot, nor did it achieve targeted structural optimization. Future research should focus on structural optimization informed by mechanical analysis to reduce printing costs, improve air permeability, reduce weight, and enhance comfort. This approach aims to minimize the orthosis's weight while ensuring adequate strength and improving overall comfort (27). The primary focus of our study was to evaluate the immediate therapeutic effectiveness of 3D-printed orthoses in improving motor function in children with SPC. As such, long-term durability and cost-effectiveness were not addressed within the scope and timeframe of this study. Regarding the materials used, we selected TPU95A for the insoles and aluminum alloy for the orthotic leg parts based on their durability, strength, and biocompatibility. These materials have been well-documented for their performance in similar applications. For example, TPU has been shown to have low thermal expansion (28), making it suitable for long-term use in environments with temperature fluctuations. Additionally, TPU is known for its high energy storage capacity and flexibility, as demonstrated in passive prosthetic designs (29). As for the aluminum alloy, studies have demonstrated its lightweight yet strong properties (30), making it an ideal material for use in orthotic devices. We believe this material choices ensure the short-term effectiveness of the orthoses, and future studies could explore their long-term durability and cost-effectiveness in a longitudinal context. Furthermore, 3D printing technology enables for the creation of complex geometric shapes, offering a high degree of design flexibility. In terms of insole design, features such as varus pads, valgus pads, and heel pads can be incorporated, similar to biomechanical insoles. These additions help to better restore the biomechanics of the lower limbs in children with CP. When integrated with ankle-foot orthoses, this approach provides a novel way to further enhance their functionality and improve patient outcomes (31).

Although the results of this study are promising, several limitations must be acknowledged. First, the relatively small sample size may limit the generalizability of the findings. Future research should involve larger cohorts and multi-center studies to enhance the external validity and confirm the broader applicability of these results. Second, given that our study was designed within a limited timeframe, the evaluation of potential complications or side effects, long-term efficacy and compliance with orthosis use is insufficient, necessitating long-term follow-up data to confirm sustained benefits. We suggest that these aspects could be explored in future research, which would provide a more comprehensive understanding of the benefits and risks of 3D-printed orthoses. Additionally, the study did not assess the specific impact of orthosis use on the quality of life of children. Furthermore, a formal, validated patient-reported outcome measure (PROM) for comfort was not included in our evaluation, which is a limitation given that one of our design goals was to provide a more comfortable orthosis. Future studies should incorporate quality of life measures to comprehensively evaluate the practical utility of orthoses.

In summary, 3D-printed AFOs demonstrate superior improvement effects compared to traditional methods in the rehabilitation of children with SPC, particularly in enhancing joint mobility and walking function. With the continuous advancement of 3D printing technology, its application in personalized medical equipment manufacturing is expected to expand further, potentially providing substantial health benefits to a broader population of patients.

## Data Availability

The raw data supporting the conclusions of this article will be made available by the authors, without undue reservation.
